# A systematic review and meta-analysis of risks and benefits with breast reduction in the public healthcare system: priorities for further research

**DOI:** 10.1186/s12893-021-01336-7

**Published:** 2021-09-11

**Authors:** Emmelie Widmark-Jensen, Susanne Bernhardsson, Maud Eriksson, Håkan Hallberg, Christian Jepsen, Lennart Jivegård, Ann Liljegren, Max Petzold, Mikael Svensson, Fredrik Wärnberg, Emma Hansson

**Affiliations:** 1 Department of Clinical Sciences, Sahlgrenska Academy, University of Gothenburg, Gröna Stråket 8, SE-413 45 Gothenburg, Sweden; 2 Department of Plastic and Reconstructive Surgery, Region Västra Götaland, Sahlgrenska University Hospital, Gröna Stråket 8, SE-413 45, Gothenburg, Sweden; 3 Region Västra Götaland, Research and Development Primary Health Care, Kungsgatan 12, SE-411 19 Gothenburg, Sweden; 4 Health Technology Assessment Centre, Region Västra Götaland, Sahlgrenska University Hospital, Röda Stråket 8, 413 45 Gothenburg, Sweden; 5 Department of Health and Rehabilitation, Institute of Neuroscience and Physiology, The Sahlgrenska Academy, University of Gothenburg, Gothenburg, Sweden; 6 Department of Surgery, Region Västra Götaland, Sahlgrenska University Hospital, Blå Stråket, 413 46 Gothenburg, Sweden; 7 Region Västra Götaland, Sahlgrenska University Hospital, Medical Library, Vita Stråket 12, SE-413 45 Gothenburg, Sweden; 8 School of Public Health and Community Medicine, Institute of Medicine, University of Gothenburg, Gothenburg, Sweden; 9 Health Metrics, The Sahlgrenska Academy, University of Gothenburg, Gothenburg, Sweden

**Keywords:** Breast reduction, Reduction mammaplasty, Breast hypertrophy, Plastic surgery, Evidence-based medicine, Prioritizing

## Abstract

**Background:**

There is no consensus for when publicly funded breast reduction is indicated and recommendations in guidelines vary greatly, indicating a lack of evidence and unequal access. The primary aim of this review was to examine risks and benefits of breast reduction to treat breast hypertrophy. Secondary aims were to examine how the studies defined breast hypertrophy and indications for a breast reduction.

**Methods:**

A systematic literature search was conducted in PubMed, MEDLINE All, Embase, the Cochrane Library, and PsycInfo. The included articles were critically appraised, and certainty of evidence was assessed using the GRADE approach. Meta-analyses were performed when possible.

**Results:**

Fifteen articles were included; eight reporting findings from four randomised controlled trials, three non-randomised controlled studies, three case series, and one qualitative study**.** Most studies had serious study limitations and problems with directness. Few of the studies defined breast hypertrophy. The studies showed significantly improved health-related quality of life and sexuality-related outcomes in patients who had undergone breast reduction compared with controls, as well as reduced depressive symptoms, levels of anxiety and pain. Most effect sizes exceeded the reported minimal important difference for the scale. Certainty of evidence for the outcomes above is low (GRADE ⊕ ⊕). Although four studies reported significantly improved physical function, the effect is uncertain (very low certainty of evidence, GRADE ⊕). None of the included studies reported data regarding work ability or sick leave. Three case series reported a 30-day mortality of zero. Reported major complications after breast reduction ranged from 2.4 to 14% and minor complications from 2.4 to 69%.

**Conclusion:**

There is a lack of high-quality studies evaluating the results of breast reduction. A breast reduction may have positive psychological and physical effects for women, but it is unclear which women benefit the most and which women should be offered a breast reduction in the public healthcare system. Several priorities for further research have been identified.

**Pre-registration:**

The study is based on a Health Technology Assessment report, pre-registered and then published on the website of The Regional HTA Centre of Region Västra Götaland, Sweden.

**Supplementary Information:**

The online version contains supplementary material available at 10.1186/s12893-021-01336-7.

## Background

Publicly funded welfare-type healthcare systems with a strong emphasis on equal access to healthcare are increasingly struggling with resource constraints. This requires a standardisation, with continuing re-evaluation, of what should be reimbursed and what should be rationed [1–3]. The nature of plastic surgery entails an element of subjectivity and studies have revealed that there is a variation in what is offered which could indicate a lack of evidence and unequal access [1, 4–7]. One procedure that has been debated, and where guidelines vary, is breast reduction due to breast hypertrophy [3, 5, 8–10].

Breast hypertrophy is a condition that may give rise to both physical and psychosocial symptoms, including muscle pain, such as back and shoulder pain, headache, postural changes, bra strap grooves, intertrigo, inability to participate in exercise and sports, sexual problems, bullying, body image problems, and problems with poorly fitting clothes [11]. Most of the symptoms described impair health-related quality of life (HRQoL). A breast reduction (reduction mammaplasty) is considered effective at reducing physical and psychosocial symptoms and improving HRQoL [12, 13]; however, similar effects are also achieved when a breast reduction is performed for cosmetic reasons and therefore it is difficult to distinguish which patients should be operated in the publicly funded healthcare system [14, 15]. Moreover, there is no standardisation regarding the assessment and prioritising of functional problems, such as back pain, compared to non-functional problems, for example suffering due to appearance [16].

There is no commonly accepted definition of breast hypertrophy and no consensus for when a breast reduction is indicated and should be reimbursed. Some guidelines define breast hypertrophy according to breast volume. For example, the current national Swedish guidelines [17] base the definition on anthropomorphic measurements of mean breast volume (405 ml, median 359 ml) in a population of randomly chosen women [18]. Hypertrophy is defined as at least twice the mean volume observed in the anthropomorphic measurement studies; that is, a volume of > 800 ml per breast. Previous Swedish studies, conducted before the guidelines were established, showed that many women who want a breast reduction have a volume of > 800 ml [19, 20]. Other definitions of breast hypertrophy include the Sacchini criteria [21] and bra size. The Sacchini criteria [21] are based on the mean measurement of the nipple to the inframammary fold distance and the nipple to the lateral border of the sternum distance. A mean distance of less than 9 cm is considered to indicate a small breast, 9–11 cm a normally sized breast and > 11 cm breast hypertrophy. When bra size is used, a cup D or larger is typically considered to indicate breast hypertrophy. In healthcare systems with third party payers, such as the United States system, insurance companies often assess the medical necessity for a breast reduction based on the amount of tissue that can be removed in a normal weight patient [9], usually according to the The Schnur Sliding Scale [10, 22].

The aim of this review was to examine the risks and benefits of breast reduction to treat breast hypertrophy. Specifically, the primary aims were to investigate whether breast reduction is better than no surgery**,** in women with symptomatic breast hypertrophy and a BMI of ≤ 35, regarding HRQoL, depressive symptoms, anxiety symptoms, sexuality-related outcomes, work ability, sick leave, physical function, pain, and patient experience, and whether it is safe. Secondary aims were to examine how the studies defined breast hypertrophy and which indications for a breast reduction were used.

## Methods

### Protocol

This is a systematic review and meta-analyses based on a Health Technology Assessment report [23]. The protocol was pre-registered on the webpage of The Regional Health Technology Assessment Centre of Region Västra Götaland, Sweden (*HTA-centrum*).

### Eligibility criteria and study selection

Studies examining risks and benefits with breast reduction in breast hypertrophy were included. Included articles had to meet criteria defined in a PICO (population, intervention, comparison, and outcome) [24] (Table 1). A patient was included in the work group when the PICO was defined.

**Table 1:** PICO

PICO	
P	Women who seek health care for symptomatic breast hypertrophy and with a BMI ≤ 35 Excluded: Women operated for breast cancer or who have had a breast augmentation
I	Breast reduction
C	C1: no treatment C2: non-surgical treatment
O	*Critical for decision-making*
	Mortality
	Complications
	Health related Quality of Life (HRQoL)
	*Important for decision-making*
	Depressive symptoms
	Anxiety symptoms
	Sexuality-related outcomes
	Work ability
	Sick leave Physical function
	Pain
	Experiences of having a breast reduction
	*Patient-reported outcomes had to be measured with validated scales*

Eligible study designs were randomised controlled trials (RCTs), non-randomised controlled studies comprising ≥ 100 patients, case series if ≥ 1000 patients were reported (only for complications), all case reports/series reporting deaths, and qualitative studies. All the authors independently assessed whether the articles met the inclusion criteria and disagreements were resolved by discussion.

### Information sources and search

In June 2020 two medical librarians (authors AL, ME) together performed a search in MEDLINE All (Ovid), PubMed, Embase (Ovid), the Cochrane Library (Wiley) and APA PsycInfo (Ebsco), using controlled vocabulary (MeSH, Emtree) such as breast hyperplasia and breast reduction and relevant free-text terms. The searches for all databases were validated by discussion and are available in Additional file 1. Reference lists of relevant articles were scrutinised for additional references. The web sites of the Swedish Agency for Health Technology Assessment and Assessment of Social Services (SBU) and the Norwegian Institute of Public Health were visited. The search was limited to English, Swedish, Norwegian and Danish languages, to human studies and publications from January 1990 to June 2020. The search was limited to this time period, as a previous systematic review has demonstrated that there is no relevant literature from before this date [17]. All articles remaining after the initial selection were obtained in full text for assessment by the other authors (EWJ, SB, HH, CJ, LJ, MP, MS, FW, EH). All authors independently assessed all the full-text articles (EWJ, SB, ME, HH, CJ, LJ, AL, MP, MS, FW, EH). Final inclusion was determined by consensus.

### Data collection process and data items

Data were extracted by one author and verified by another. Information collected included: first author, year of publication, study country, study design, study scope, number of patients and controls, dropouts, study groups, body mass index (BMI), age, tobacco use, definition of breast hypertrophy, resection weight and outcomes according to the PICO.

### Statistical analysis

The results of each article were tabulated per outcome (Tables 2, 3, 4, 5, 6, 7). When possible, data were pooled and subjected to meta-analysis using Review Manager (RevMan) and the Metan-command in Stata version 16. Random effects model using the method of DerSimonian and Laird, with the estimate of heterogeneity being taken from the inverse-variance fixed-effect model, was used. When only median and range was reported in the original studies, median was used as a proxy for mean and range divided by 6 was used a proxy to SD since mean ± 3*SD covers about 99.7% of the population values. If SD was only reported for baseline this value was also used for follow-up. For most outcomes, meta-analysis was not possible due to heterogeneity in measures and follow-up time.

**Table 2:** Characteristics of included studies

Author Year Country	Study design	Study duration (years) Follow-up (mean number of months)	Study groups; Intervention and control treatment	Patients (n)	Mean age (years)	Mean BMI	Smokers (n, %)	Definition of breast hypertrophy	Resection weight, g (mean)
Araujo 2014 Brazil [40]	RCT (cost-utility)	SD: NR FU: 6	I: Breast reduction; conventional technique (inverted T-shaped scar and medial pedicle technique in most patients) C1: No treatment	60 I: 30 C: 30 BMI >30 excluded)	I:32 C:35.5 (median)	I: 26.4 C: 26.3 (median)	NR	Classification by Sacchini et al	1200
Beraldo 2016 Brazil [41]	RCT (same RCT as above)	As above	As above	As above	As above	As above	NR	As above	As above
Freire 2007 Brazil [37]	RCT	SD: NR FU: 6	I: Breast reduction; rigid outlining, transferring to opposite breast, preservation of papillary-areola complex using fatty dermal pedicle. Inverted T-shaped scar C1: Waiting list for reduction mammaplasty 6 months later	100 I: 50 C: 50 BMI> 30 excluded)	31.95	25.56	0	NR	1052.19
Neto 2008 Brazil [36]	RCT (same RCT as above)	SD: 2.08 FU: 6	As above	As above	As above	As above	0	NR	As above
Iwuagwu 2006 UK [39]	RCT	SD: 1.67 FU: 4	I: Bilateral breast reduction with an inferior pedicle C2: Physiotherapist-instructed upper body exercise 3 times/week while on wait list for surgery	73 I: 36 C: 37 No restriction related to BMI	39.15	28.5	NR	Bra cup size E or more in conjunction with symptoms in the upper body associated with mammary hypertrophy	NR
Iwuagwu 2006 UK [31]	RCT (same RCT as above)	As above	As above	73 I: 40 C: 73	As above	As above	NR	As above	NR
Saarinemi 2008 Finland [34]	RCT	SD: NR FU: 6.35	I: Breast reduction C1: Waiting list	82 I: 40 C: 42 No restriction related to BMI	46.35	29.65	NR	NR	670
Saarinemi 2009 Finland [57]	RCT (same RCT as above)	As above	As above	As above	As above	As above	NR	NR	As above
Andrade 2018 Brazil [42]	Non-randomized controlled study	SD: 1 FU: 6–12	I: Breast reduction C1: Waiting list	100 I: 50 C: 50 BMI < 30	I: 33 C: 31 (median)	I: 27 C: 26 (median)	NR	“By the criteria by Sacchini et al. and Franco & Rebello”	1107
Hermans 2005 Netherlands [43]	Non-randomized controlled study	SD: 2 FU: 25.4 (mean, intervention group)	I: Breast reduction, modified Strömbäck procedure with mediocranial pedicle C1: Waiting list	165 I:94 C:71 BMI < 30	37.3	25.65	NR	Cup size D or above	536
Janik 2019 Poland [62]	Non-randomized controlled study	SD: 0.25 months FU: 23.56 (mean)	I: Breast reduction C1: Waiting list	102 I:75 C: 27	38	27.5	24%	NR	NR
Fairchild 2020 USA [32]	Case series	SD: 7 FU: 1	I: Breast reduction	283 (not obese, (BMI < 30);Not included because BMI < 35 not separately reported: 259 (obese, BMI > 30, range 32–38)	17 (median)	26 (median)	NR	NR	NR
Nelson 2014 USA [33]	Case series	SD; 7 FU: 1	I: Breast reduction	2074 (BMI < 30); 1308 (BMI 30–34.9)	NR	NR	10%	NR	NR
Simpson 2019 USA [49]	Case series	SD: 10 FU: 1	I: Breast reduction	8180 (BMI < 30); 4656 (BMI 30.1–35)	NR (solely reported for total cohort)	NR (solely reported for total cohort)	NR	NR	NR
Shakespeare 1999 UK [38]	Qualitative	SD: 2,75 FU: 24	I: Breast reduction	110	35	NR	NR	NR	NR

*RCT* randomised controlled trial, *NR* not reported, *SD* study duration, *FU* follow-up, *BMI* body mass index, *HRQoL* health-related quality of life

**Table 3:** Health-related quality of life

Author Year Country	Study design	Number of patients	Withdrawals—dropouts	Intervention Breast reduction Mean (SD)	Control No surgery Mean (SD) P values of intergroup difference if not state otherwise	Comments	Directness*	Study limitations*	Precision*
Araujo 2014 Brazil [40]	RCT	*60* I: 30 C: 30	*2* 1: 0 C: 2	*SF-6D* Median (range) Preop: 0.61 (0.45–0.83) 3 m: 0.75 (0.50–0.97) 6 m: 0.76 (0.44–0.97)	*SF-6D* Median (range) Preop: 0.61 (0.39–0.835) 3 m: 0.65 (0.43–0.83), p < 0.001 6 m: 0.63 (0.42–0.88) p = 0.008	*SF-6D*: Short Form 6 Dimensions questionnaire. Scale 0.29–1.00 (1.00 denotes perfect health). Minimal important difference (MID) for SF-6D has been suggested to be in the range of 0.01 to 0.10 [35] (SF-36 measured but not reported)	?	?	?
Iwuagwu 2006 UK [31]	RCT	*73* I: 36 C: 37	*0* I: 0 C: 0	*FANLT* Physical well-being: 24.2 (3.7) Social well-being: 24.6 (4.7) Emotional well-being: 17.3 (3.7) Functional well-being: 23.3 (5.2) *SF-36* Physical component score: 50.0 (7.5) Mental component score: 53.2 (8.8) *EQ-5D* Self-care: 1.03 (0.18) Activities: 1.32 (0.61) Pain: 1.46 (0.57) Anxiety and depression: 1.21 (0.50)	*FANLT* Physical well-being: 19.2 (5.1) p < 0.001 Social well-being: 20.7 (4.7) p = 0.002 Emotional well-being: 13.0 (3.5) p < 0.001 Functional well-being: 17.1 (6.2) p < 0.001 *SF-36* Physical component score: 42.0 (9.6) p < 0.001 Mental component score: 42.0 (11.1) p < 0.001 *EQ-5D* Self-care: 1.05 (0.23) p = 0.978 Activities: 1.48 (0.51) p = 0.061 Pain: 2.05 (0.52) p < 0.001 Anxiety and depression: 1.59 (0.55) p = 0.006	*FANLT:* Functional Assessment of Non-Life-Threatening Conditions (higher score = better health, range not stated) *SF-36*: Short Form-36 Health survey (36 items, range 0–100, higher score = better health) EQ-5D (EuroQol): The European Quality of Life-5 Dimensions (mobility, self-care, usual activities, pain/discomfort, anxiety/depression). Range 1–3, lower score = better. 4 dimensions reported in article All are validated HRQoL tools Control group underwent physiotherapy	?	?	?
Saariniemi 2008 Finland [34]	RCT	*82* I: 40 C: 42	*18* I: 11 C: 7	*SF-36* Utility index score (SF-6D): 0.820 (SD NR) Physical component score: 51.7 (SD NR) Mental component score: 53.8 (SD NR) *15D index score* 0.917 (SD NR)	*SF-36* Utility index score (SF-6D): 0.663 (SD NR) MD 0.157 (95% CI 0.107 to 0.220) p < 0.0001 Physical component score: 43.3 (SD NR) MD 8.4 (95% CI 5.8 to 11.8) p < 0.0001 Mental component score: 46.2 (SD NR) MD 7.6 (95% CI 3.2 to 13.1) p < 0.002 *15D index score* 0.861 (SD NR) MD 0.056 (95% CI 0.041 to 0.103), p < 0.0001	*SF-36:* Short Form-36 Health survey (range 0–100, higher score = better health) *SF-6D:* Single health utility index score. Part of SF-36 (range 0.29–1.00, higher score = higher function) *15D*: Finish QoL questionnaire (higher score = better health, range 0–1)	?/ +	?	+
Andrade 2018 Brazil [42]	Non-randomized controlled study	*100* I: 50 C: 50	NR	*Breast-Q* Median (range) Satisfaction with breasts: 70 (30–100) Psychosocial well-being: 92 (0–100) Sexual well-being: 88 (21–100) Physical well-being: 79 (48–100)	*Breast-Q* Median (range) Satisfaction with breasts: 23 (0–50) p = 0.001 Psychosocial well-being: 33 (0–71) p = 0.001 Sexual well-being: 29 (0–78) p = 0.001 Physical well-being: 48 (0–83) p = 0.001	*Breast-Q:* Total scores ranging from 0–100. Higher score indicates greater satisfaction or better quality of life	+	−	?/ +
Hermans 2005 Netherlands [43]	Non-randomized controlled study	*165* I: 94 C: 71	*10* I: 10 C: 0	*SF-36* Physical function: 84.76 Pain: 77.65 Vitality: 67.01 Social activities: 83.69 Emotional status: 80.95 Mental health: 75.22 Physical activities: 76.19 Health perceptions: 72.26 *EQ-5D* *Pain:* No problems: 51.2% Some problems: 44.0% Many problems: 4.8% *Daily activities:* No problems: 72.3% Some problems: 25.3% Many problems: 2.4%	*SF-36* Physical function: 77.46, p < 0.05 Pain: 57.00, p < 0.001 Vitality: 56.83, p < 0.01 Social activities: 68.30, p < 0.001 Emotional status: 64.32, p < 0.01 Mental health: 66.42, p < 0.01 Physical activities: 65.85, p = NR Health perceptions: 65.42, p < 0.05 *EQ-5D* *Pain:* No problems: 14.1%, p < 0.001 Some problems: 78.9% Many problems: 7.0% *Daily activities:* No problems: 46.5%, p < 0.005 Some problems: 50.7% Many problems: 2.8%	*SF-36:* Short Form-36 Health survey (higher score = better health, range 0–100) *EQ-5D:* The European Quality of Life-5 Dimensions (mobility, self-care, usual activities, pain/discomfort, anxiety/depression). 3 answers possible within each dimension. Only 2 dimensions stated in article	?	?/−	?

* + No or minor problems; ? Some problems; - Major problems

*SF-6D* Short Form 6 Dimensions questionnaire Scale, *FANLT* Functional Assessment of Non-Life-Threatening Conditions, *SF-36* Short Form-36 Health survey, *MD* Mean difference, *15D* Finish QoL questionnaire, *EQ-5D* The European Quality of Life-5 Dimensions, *NR* not reported, *SD* standard deviation

**Table 4:** Depression and anxiety

Author Year Country	Study design	Number of patients	Withdrawals—dropouts	Intervention Breast reduction Mean (SD)	Control No surgery Mean (SD) P values of intergroup difference if not state otherwise	Comments	Directness*	Study limitations*	Precision*
Beraldo 2016 Brazil [41]	RCT	I: 30 C: 30	I: 1 C: 3	*Depression score (BDI)* Baseline: 12.4 (9.0) 3 months: 10.2 (9.9) 6 months: 7.2 (9.9) Intragroup change: Baseline to 3 and 6 months p < 0.001	*Depression score (BDI)* Baseline: 13.2 (9.6) p = 0.89 3 months: 13.0 (8.5) p = 0.12 6 months: 13.7 (10.5) p = 0.01 Intragroup change: Baseline to 3 and 6 months p = 0.89	Beck Depression Inventory (BDI) (21 items, range 0–63, higher score indicates worse depression) < 10 = no or minimal depression 10–16 = mild depression 17–29 = moderate depression 30–63 = severe depression A MID of 17.5% of the total score (11 points) has been suggested for BDI [63]	?	?	?
Iwuagwu 2006 UK [39]	RCT	I: 36 C: 37	0	*Depression score* Baseline: 0.69 (0.30) 4 months: 0.39 (0.27) *Proportion depressed (no. (%)):* Baseline: Normal score: 28 (78) Borderline score: 6 (17) Abnormal score: 2 (6) 4 months: Normal score: 34 (94) Borderline score: 1 (3) Abnormal score: 1 (3)	*Depression score* Baseline: 0.70 (0.29) 4 months: 0.79 (0.27) p < 0.001 *Proportion depressed (no. (%)):* Baseline: Normal score: 27 (73) Borderline score: 8 (22) Abnormal score: 2 (6) 4 months: Normal score: 25 (67) Borderline score: 10 (27) Abnormal score: 2 (6) p < 0.001	Hospital Anxiety and Depression Scale (HADS) (7 items, range 0–21. Higher score indicates worse depression) 0–7 ‘normal’ 8–10 ‘borderline’ ≥ 11 ‘clinical depression/anxiety’ Depression scores were transformed to appropriate a Gaussian distribution (1 + log 10) A MID of 1.7 has been suggested for HADS [64]	?	?	?
				*Anxiety score* Baseline: 9.1 (3.9) 4 months: 5.0 (3.5) Baseline: No (%) Normal score: 12 (33) Borderline score: 11 (31) Abnormal score: 13 (36)	*Anxiety score* Baseline: 9.1 (4.0) 4 months: 9.6 (3.8) p < 0.001 Baseline: No (%) Normal score: 12 (32) Borderline score: 11 (30) Abnormal score: 14 (38)				
				4 months: Normal score: 30 (83) Borderline score: 4 (11) Abnormal score: 2 (6)	4 months: Normal score: 10 (28) Borderline score: 10 (28) Abnormal score: 17 (47) p < 0.001 MD 4.6				
Saariniemi 2009 Finland [57]	RCT	I: 40 C: 42	I: 11 C: 7	*RBDI* *Depression* Baseline: 5 (2.5–6.5) 6 months: 0 (0.0–2.5) Median (interquartile)	*RBDI* *Depression* Baseline: 4 (1.0–8.0) 6 months: 4 (0.0–7.0) p < 0.01 Median (interquartile)	RBDI: Raitasalo’s modification of the short form of the Beck Depression inventory (range 0–39, lower better) 5–7: mild depression 8–15: moderate depression > 16: severe depression	?/ +	?	+
				*Proportion depressed (no. (%)):* Baseline: 16 (55) 6 months: 2 (7)	*Proportion depressed (no. (%)):* Baseline: 15 (43) 6 months: 15 (43) p < 0.01	*Proportions:* Depressed = RBDI depression score > 4			
				*Anxiety* No. (%) Baseline: 18 (62) 6 months: 3 (10)	*Anxiety* No. (%) Baseline: 18 (51) 6 months: 12 (34) p = 0.04 MD 9				

* + No or minor problems; ? Some problems; - Major problems

*BDI* Beck Depression Inventory, *HADS* Hospital Anxiety and Depression Scale, *RBDI* Raitasalo’s modification of the short form of the Beck Depression Inventory

**Table 5:** Sexually-related outcomes

Author Year Country	Study design	Number of patients	Withdrawals—dropouts	Intervention Breast reduction Mean (SD)	Control No surgery Mean (SD) P values of intergroup difference if not state otherwise	Comments	Directness*	Study limitations*	Precision*
Beraldo 2016 Brazil [41]	RCT	I: 30 C: 30	I: 1 C: 3	*Sexual function* Baseline: 24.7 (8.8) 6 months: 27.5 (6.9)	*Sexual function* Baseline: 23.9 (9.6) p = 0.96 6 months: 22.5 (9.3) p < 0.001 MD 5.0	Female Sexual Function Index (FSFI). The questionnaire includes 19 questions on sexual activity during the last 4 weeks. It has 6 domains: desire, arousal, lubrication, orgasm, satisfaction, and discomfort/pain. A higher score means a better function. A total score of 26.55 or less indicates sexual dysfunction A MID of 4.2 has been suggested for FSFI [65]	?	?	?
Andrade 2018 Brazil [42]	Non-randomized controlled study	I: 50 C: 50	NR	*Sexual well-being* 6 months-1 year: 88 (21–100) median (range)	*Sexual well-being* 29 (0–78) median (range) p = 0.001 MD 66	Sexual well-being domain of BREAST-Q (reduction/ mastopexy module) Score 0–100, a higher score means better outcome Baseline values are not given	+	-	?/ +
Janik 2019 Poland [62]	Non-randomized controlled study	I: 75 C: 27	NR	*Sexual quality of life* 12–36 months: 76.7 (11.6) (mean follow-up 23.6 months)	*Sexual quality of life* 64.4 (13.7) p < 0.01 MD 12	Sexual Quality of Life-Female (SQoL-F): 18 items, each scored from 1–6, total score 18–108 Higher score better	+	-	-
				*Sexual function* 12–36 months: 27.4 (9.1)	*Sexual function* Pre-operative: 21 (11.4) p = 0.03	Female Sexual Function Index (FSFI). Higher score better			
				*Sexual well-being* 12–36 months: 72 (14)	*Sexual well-being* Pre-operative: 39.3 (14.5) p < 0.01	Sexual well-being domain of BREAST-Q (reduction/ mastopexy module). Baseline values not reported			

* + No or minor problems; ? Some problems; - Major problems

**Table 6:** Physical function

Author Year Country	Study design	Number of patients	Withdrawals—dropouts	Intervention Breast reduction Mean (SD)	Control No surgery Mean (SD) P values of intergroup difference if not state otherwise	Comments	Directness*	Study limitations*	Precision*
Freire 2007 Brazil [37]	RCT	100 I: 50 C: 50	8 I: 4 C: 4	*HAQ-20* Pre-op: 0.44 (0.38) 6 months post-op: 0.12 (0.23)	*HAQ-20* Baseline: 0.48 (0.40) 6 months after baseline: 0.46 (0.30) p < 0.001 MD 0.34	HAQ-20 has 8 dimensions that evaluate aspects of daily life: dress, get up without support, feed yourself, walk on the flat, take a shower, reach objects, grasp objects, domestic tasks. It gives a summary score between 0 (able) to 3 (disabled)	+	?/−	?
Neto 2008 Brazil [36]	RCT (same as above)	100 I: 50 C: 50	8 I: 4 C: 4	*Roland-Morris questionnaire* Pre-op: 5.9 (4.9) 6 months post-op: 1.2 (1.9) Intragroup difference, p < 0.001	*Roland-Morris questionnaire* Baseline: 6.2 (4.8) 6 months after baseline: 6.2 (3.9) Intragroup difference, N.S MD 0.5 (p < 0.001)	Roland-Morris questionnaire measures functional capacity and is scored from 0 (best performance) to 24 (worst performance) Intergroup difference NR	+	−	−
Saariniemi 2008 Finland [34]	RCT	82 I: 40 C: 42	8 I: 11 C: 7	*SF-36 physical summary score* Pre-op: 42 (8.6) 6 months post-op: 51.7 (SD NR)	*SF-36 physical summary score* Baseline: 42.6 (8.9) 6 months after baseline: 43.3 (SD NR) MD 8.4 (95% CI 5.8 to 11.8), p < 0.0001	The SF-36 physical summary score represents a norm-based scoring with a mean value of 50 and a SD of 10 (range 0–100). The higher the score, the greater the satisfaction. No MID is established	?/ +	?	+
Andrade 2018 Brazil [42]	Non-randomized controlled study	100 I: 50 C: 50	0	*Breast Q physical well-being* Post-op median: 79 (48–100)	*Breast Q physical well-being* pre-op median: 48 (0–83) p = 0.001	Total scores for the subscale physical well-being range from 0 to 100. The higher the score, the greater the satisfaction. There are no MID for subscales	+	−	?/ +
Hermans 2005 The Netherlands [43]	Non-randomized controlled study	165 I: 94 C: 71	10 I: 10 C: 0	*SF-36 physical function* Postop mean value after 12–24 months: 84.76	*SF-36 physical function* Before surgery 77.46 p < 0.05	SF-36 is scored 0–100 where a higher score indicates better health status. MID not possible to establish	?	?/−	?
				*EQ 5D-daily activities* Postop mean after 12–24 months: No problems 72.3 Some problems 25.3 Many problems 2.4	*EQ 5D-daily activities* Before surgery No problems 46.5 Some problems 50.7 Many problems 2.8 p < 0.005				
				*DAS-59 I have physical disabilities because of my features* 12–24 months postoperatively: Almost never 89% Sometimes 6% Always often 5%	*DAS-59 I have physical disabilities because of my features* Almost never 6% Sometimes 23% Always often 72% p = NS				

*DAS-59* Derriford Appearance Scale 59, *EQ-5D* EuroQol 5 Dimensions, *HAQ-20* Stanford Health Assessment Questionnaire, *MID* minimally clinically important difference, *MD* mean difference, *NR* not reported, *NS* non-significant, *SF36* Short Form Health Survey 36

^*^ + No or minor problems; ? Some problems; - Major problems

**Table 7:** Pain

Author Year Country	Study design	Number of patients	Withdrawals-dropouts	Intervention Breast reduction Mean (SD)	Control No surgery Mean (SD) P values denote intergroup difference if not otherwise depicted	Comments	Directness*	Study limitations*	Precision*
Iwuagwu 2006 UK [31]	RCT	*73* I: 36 C: 37	*0*	*EQ-5D* Pain: Baseline: 1.88 (0.46) 4 months: 1.46 (0.57)	*EQ-5D* Pain: Baseline: 1.94 (0.52) 4 months: 2.05 (0.52) p < 0.001 MD 0.59	*EuroQol EQ-5D*: The European Quality of Life-5 Dimensions Pain assessment scores are part of the questionnaire. Lower score = less pain, range 1–3	?	?	?
Saariniemi 2008 Finland [34]	RCT	82 I: 40 C: 42	18 I: 11 C: 7	*FBAS* 11.8 (SD NR)	*FBAS* 57.9 (SD NR) MD − 46.1 (95% CI − 49.8 to − 40.7), p < 0.0001	*FBAS:* Finnish Breast Associated Symptoms questionnaire (Higher scores = more symptoms, range 0–100)	?/ +	?	+
				*FPQ* 7.0 (SD NR)	*FPQ* 26.5 (SD NR) MD − 19.5 (95% CI − 25.2 to − 14.3), p < 0.0001	*FPQ:* Finnish Pain Questionnaire (Higher scores = more pain, range 0–100)			
Freire 2007 Brazil [37]	RCT	100 I: 50 C: 50	8 I: 4 C: 4	*VAS:* *Lower back* Baseline: 5.7 (2.7) 6 months: 1.3 (2.5)	*VAS:* *Lower back pain* Baseline: 6.0 (3.3) 6 months: 5.3 (2.8) p < 0.001	*VAS:* Visual analogue scale (0: No pain, 10: Intense pain Same cohort as Neto et al. [36]. Lower back pain data identical	+	?/−	?
				*Shoulders* Baseline: 6.1 (2.7) 6 months: 1.1 (1.8) p < 0.001 (intragroup difference)	*Shoulders* Baseline: 6.2 (3.2) 6 months: 6.9 (2.6) p < 0.001 NS (intragroup difference)	A MID of 0.9 has been suggested for VAS [66]			
				*Neck* Baseline: 5.2 (2.9) 6 months: 0.9 (1.3) p < 0.001 (intragroup difference)	*Neck* Baseline: 4.7 (3.6) 6 months: 5.1 (3.1) p < 0.001 NS (intragroup difference)				
Hermans 2005 Netherlands [43]	Non-randomized controlled study	165 I: 94 C: 71	10 I: 10 C: 0	*SF-36* Pain: 77.65	*SF-36* Pain: 57.00 p < 0.001 MD 21	*SF-36*: Short Form-36 Health survey (higher score = better health, range 0–100)	?	?/−	?
				*EQ-5D* Pain: No problems: 51.2% Some problems: 44.0% Many problems: 4.8%	*EQ-5D* Pain: No problems: 14.1% Some problems: 78.9% Many problems: 7.0% p < 0.001	*EQ-5D:* The European Quality of Life-5 Dimensions (mobility, self-care, usual activities, pain/discomfort, anxiety/depression). Only 2 dimensions stated in article			
				*DAS-59* Pain: Almost never: 68% Sometimes: 22% Always/Often: 10%	*DAS-59* Pain: Almost never: 2% Sometimes: 18% Always/Often: 80% p < 0.001	*DAS-59:* Derriford Appearance Scale 59. Higher scores indicate greater problems Pain scores were all part of QoL-questionnaires			

*NR* not reported, *NS* no significance, *EQ-5D* The European Quality of Life-5 Dimensions, *FBAS* Finnish Breast Associated Symptoms questionnaire, *FPQ* Finnish Pain Questionnaire, *VAS* visual analogue scale, *SF-36* Short Form-36 Health survey, *DAS-59* Derriford Appearance Scale 59

* + No or minor problems; ? Some problems; - Major problems

### Risk of bias in individual studies and across studies

All included randomised and non-randomised controlled studies, as well as the qualitative study, were assessed regarding directness, risk of bias and precision, as described by the GRADE working group [25–28]. Checklists for assessing study quality, modified from the Swedish Agency for Health Technology Assessment and Assessment of Social Services (SBU) [29], were used. Certainty of evidence was assessed using the GRADE approach, as very low (GRADE ⊕), low (GRADE ⊕ ⊕), moderate (GRADE ⊕  ⊕ ⊕), and high (GRADE ⊕  ⊕  ⊕ ⊕) [30]. High quality is defined as ‘further research is very unlikely to change our confidence in the estimate of the effect’, moderate quality as ‘further research is likely to have an important impact on our confidence in the estimate of effect and may change the estimate’, low as ‘further research is very likely to have an important impact on our confidence in the estimate of effect and is likely to change the estimate’, and very low as ‘any estimate of effect is very uncertain’ [30].

## Results

### Study selection

**Fig. 1:**
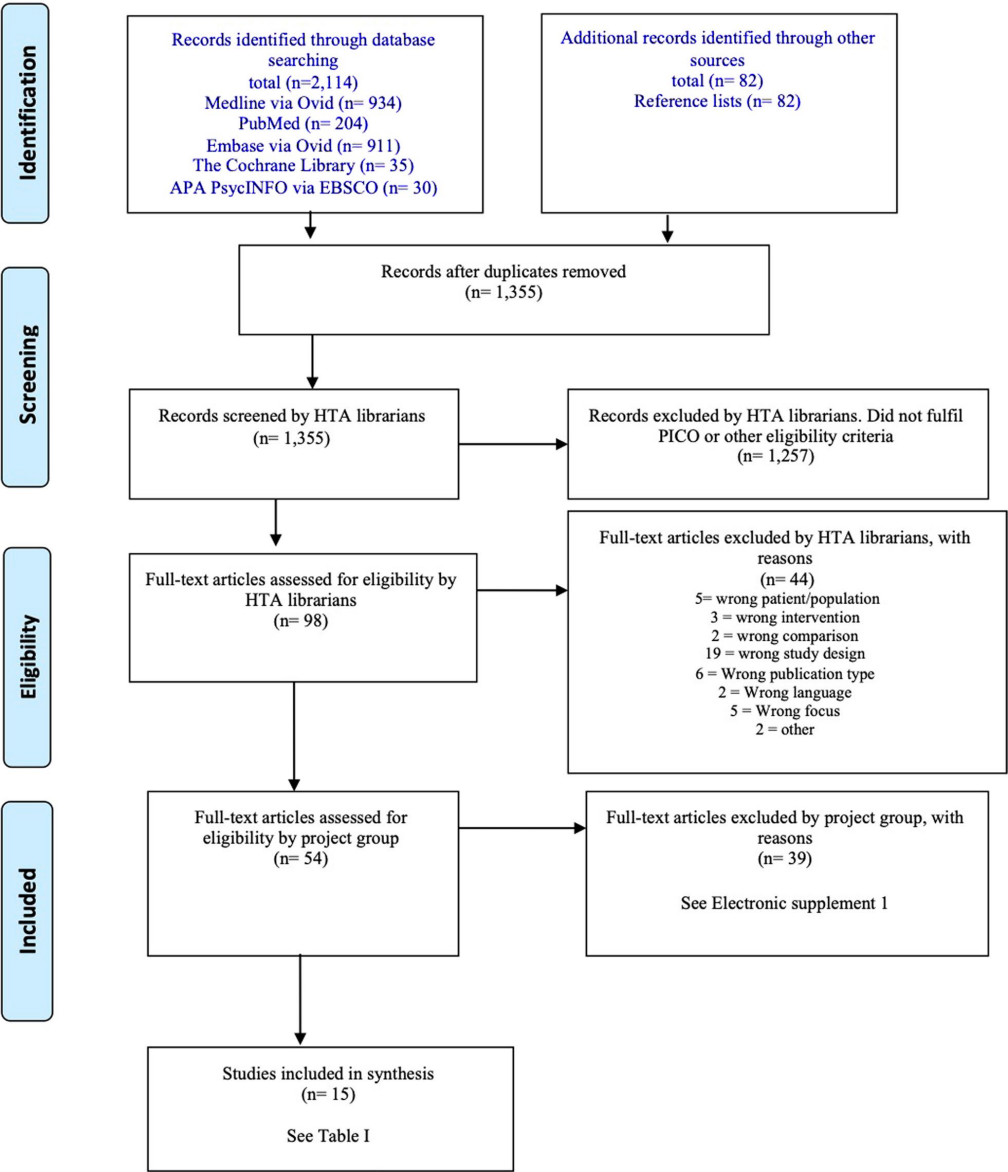
PRISMA flow diagram

The literature search identified 1355 articles after removal of duplicates. Of these, 1257 articles were excluded after screening of abstracts. Another 44 articles were excluded when they had been read in full text (Fig. 1). The 54 full-text articles left after this first selection were sent to all authors, and 15 articles were finally included in the review (Table 2). The excluded articles, with reasons for exclusion, are presented in Additional file 2.

### Study characteristics

Of the fifteen included articles, four were RCTs (reported in eight papers), three were non-randomised controlled studies, three were case series, and one was a qualitative study (Table 2). The majority of the included studies compared surgical intervention with no treatment (C1) and one study [31] with physiotherapy (C2, non-surgical treatment).

### Risk of bias within and across studies

The RCTs had serious study limitations, indirectness, and/or imprecision. Methodological issues included unclear definition of breast hypertrophy, short follow-up, lack of blinding of patients or surgeons, control groups composed of patients waiting for a breast reduction, and a lack of inter-group comparisons. Effects were measured using validated patient-reported outcome measures. The non-randomised controlled studies had some study limitations in terms of poor evaluation of potential confounding, adherence, dropouts, and unclear definitions of breast hypertrophy. The qualitative study was assessed as being of moderate quality.

### Mortality and complications

Mortality was reported in three case series (n = 104,565), all based on the same registry, NSQIP; thus, slightly overlapping. One death was reported in the population with BMI > 30, not included in this review [32]. Three RCTs, one non-randomised controlled study and three cases series, based on register data, reported surgical complications (Additional file 3). Reporting standards were heterogeneous, as complications were not predefined, and no information was given about when, how, or by whom they were diagnosed. The reported frequencies of major complications after breast reduction, such as venous thromboembolism [33, 34], varied from 2.4 to 14%, and frequencies of minor complications, such as surgical site infection and delayed wound healing, from 2.4 to 69%. Two of the included studies showed that increased BMI was a risk factor for complications [32, 33].

### Health-related quality of life

**Fig. 2:**
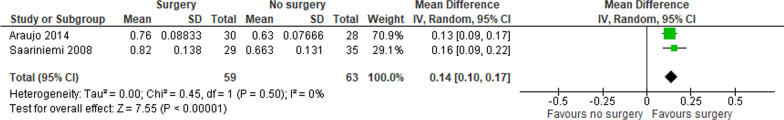
Meta-analysis of studies comparing reduction mammoplasty with no surgery using SF-6D (Health utility index score)

**Fig. 3:**
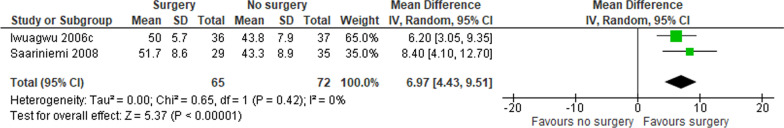
Meta-analysis of studies comparing reduction mammoplasty with no surgery, using SF-36 (Physical summary score)

**Fig. 4:**
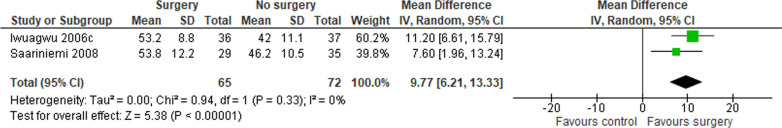
Meta-analysis of studies comparing reduction mammoplasty with no surgery, using SF-36 (Mental summary score)

Health-related quality of life was reported in three RCTs and two non-randomised controlled studies, using both generic (SF-6D, and SF-36) and disease-specific (BREAST-Q) questionnaires (Table 3). HRQoL was improved after breast reduction in all included studies, compared with no surgery. Meta-analyses (Figs. 2, 3, 4) performed for SF-6D and SF-36 scores, showed a weighted mean difference for SF-6D of 0.14 (95% CI 0.10–0.17) 6 months after surgery, implying a clinically relevant difference in HRQoL, compared with the previously suggested minimal important difference (MID) [35] (Table 3). In summary, breast reduction compared with no surgery may result in a clinically relevant improvement in HRQoL in women with breast hypertrophy (low certainty of evidence, GRADE ⊕ ⊕).

### Depression and anxiety

Depressive symptoms were reported in three RCTs (n = 215) and symptoms of anxiety in two RCTs (n = 155), using different validated assessment tools and scores (Table 4). Postoperative (4–6 months) depressive symptom rates were consistently lower in women undergoing breast reduction compared with no treatment or physiotherapy. The postoperative anxiety symptoms were measured after four to six months and were significantly lower in women who had undergone breast reduction in both studies. In summary, breast reduction, compared with no surgery, may result in a clinically relevant reduction in depressive and anxiety symptoms, in women with breast hypertrophy (low certainty of evidence, GRADE ⊕ ⊕).

### Sexuality-related outcomes

Sexuality-related outcomes were reported in one RCT and two non-randomised controlled studies (n = 262), using different instruments (Table 5). Sexual function, sexual well-being, and sexual quality of life were significantly improved after breast reduction compared with no surgery. In summary, sexuality-related outcomes may be significantly improved by breast reduction, compared with no surgical intervention (low certainty of evidence, GRADE ⊕ ⊕).

### Work ability and sick leave

Work ability and sick leave were not reported in any of the included studies.

### Physical function

Physical function after breast reduction compared with no surgery, was reported in two RCTs and two non-randomised controlled studies (n = 447) (Table 6). One RCT reported physical function in two papers [36, 37]. Statistically significant improvement in physical function after surgery was reported in the RCTs, with a follow-up time of 6 months. Significant intergroup improvement was reported in the non-randomised controlled studies regarding physical wellbeing, physical function and daily activities after surgery. In conclusion, it is uncertain whether breast reduction compared with no surgery affects physical function in women with breast hypertrophy (very low certainty of evidence, GRADE ⊕).

### Pain

Three RCTs and one non-randomised controlled study (n = 420) reported pain (Table 7), measured with different instruments. Pain was significantly reduced in all studies. In summary, breast reduction compared with no surgery may result in a clinically relevant reduction of pain in women with breast hypertrophy (low certainty of evidence, GRADE ⊕ ⊕)..

### Patient experiences of a breast reconstruction

One qualitative study including 50 patients was identified [38]. Most of the patients reported an increased physical activity after the operation and believed that the operation had changed their lives to the better. Nonetheless, a few patients reported a deterioration in self-image and quality of life and one patient expressed regret. Some patients were unsatisfied or distressed with the scarring (Additional file 4).

### Definitions of breast hypertrophy and indications for a breast reduction

As regards definitions, three studies used the Sacchini criteria, two studies used bra cup size, and 10 studies did not report how they defined breast hypertrophy (Table 2). One study reported that a bra size of E or more in combination with ‘symptoms in the upper body associated with mammary hypertrophy’ constituted an indication for surgery [39]. None of the other studies specifically reported indications for a breast hypertrophy.

## Discussion

The aim of this review was to examine the risks and benefits of breast reduction in women with breast hypertrophy, with an underlying focus on identifying specific indications for surgery in the public healthcare system.

### Methodological limitations of the included studies

Several methodological limitations were identified in all included studies. Main issues included a lack of, or the use of non-validated, definitions of breast hypertrophy and of complications, a potentially biased control group, lack of blinding, a short follow-up, and insufficient reporting of inter-group results.

The main problem with the lack of definitions of breast hypertrophy and indications for breast reductions in the studies is that it is difficult to evaluate effects of treatment when the condition is not adequately defined. Moreover, not all of the studies reported the resected amount of breast tissue, further complicating the evaluation of the effects of the intervention in relation to the severity of breast hypertrophy. There are a number of limitations regarding the use of unvalidated breast measurements, such as bra size and the Sacchini criteria, that were used in the few studies [39–43] in this review that reported their definition. Firstly, there are no conclusive studies determining what volume/weight, in relation to body build, that gives rise to physical and/or psychosocial symptoms, and symptom relief does not seem to be correlated to the amount of tissue resected [22, 44]. Secondly, the relationship between breast volume and breast weight is not clear-cut as different breasts have different density. The ratio between adipose tissue and breast tissue varies according to genetics and hormonal status and breast tissue weighs more than adipose tissue. Thirdly, breast size measurements are uncertain [45, 46]. As regards, the use of cup sizes, they are not standardised; for example, one brand’s D cup might equal another brand’s C cup. The cup size is often based on the difference in breast circumference and rib cage circumference; that is, a difference of one inch (2.54 cm) constitutes an A cup, two inches a B cup, etc. and consequently the actual volume of the cup is substantially different depending on the circumference of the rib cage. Moreover, the model of the bra, for example if it covers the entire or only part of the breast, creates different ‘volumes’. Finally, there is a considerable difference in how women want their bra to fit; that is, women with identical breast volume might wear different bra sizes [47]. In brief, it is unclear which conditions have actually been treated in the included studies.

None of the included studies stated how complications were defined and whether they had been registered in a systematic and prospective fashion or not. Similar methodological problems have been seen previously in studies on breast reduction, where most studies only register surgical site complications in an undefined way leading to an underestimation of overall complication rates [48]. In one of few publications [48] on breast reduction where complications were classified according to a validated system, Clavien–Dindo, the complication frequency was 63%, albeit retrospectively registered. A prospective approach could give an even higher complication frequency. The most common type of complication (46%) was wound healing complications [48]. The study by Winter and associates [48] was not included in this review as the number of reported patients were 486, and the inclusion requirement of > 1000 patients for case series was therefore not met. In this review, the lower complication rates are from publications reporting figures from the NSQIP registry [32, 33, 49]. In the registry, wound complications are defined as ‘superficial infection, deep wound infection, deep or organ space infection, and wound dehiscence’ [49]. In Winter et al.’s study [48], the rate of such wound complications was 9%, and the rate of milder wound complications, not requiring an intervention, such as antibiotics or debridement, was 48%. Indeed, the studies included in this report with higher complication rates seem to have included all types of wound complications. Hence, complications are common but reported frequencies are dependent on how complications are defined and classified, explaining the wide range of frequencies observed in the present review.

In all included RCTs, patients who wanted a breast reduction were randomised either to breast reduction or to a waiting list for such surgery. Therefore, all the patients were likely biased towards a wish for a breast reduction and all the controls knew that they would receive a breast reduction eventually. It can be discussed whether such patients represent an adequate untreated control group. The practice also implies that neither the patients nor the surgeons were blinded.

Another issue limiting the directness of the results, is the short follow-up time in the included studies. According to basic plastic surgical principles, a final result can never be evaluated before at least a year has passed [50]. Most of the included studies had a follow-up time of less than one year, and therefore the measured effects might not represent the final outcome of surgery. Patients who are treated with surgery they have requested themselves, initially experience a positive effect of the surgery that might diminish over time [51]. Moreover, two of the four RCTs were conducted in the same country which might limit the generalisability of the results as cultural norms [52], and perceived need for breast reduction, might be different in other parts of the world.

### Discussion of current evidence

Our review shows that complications are frequent after breast reduction. None of the included studies specifically reported the impact of different breast volumes on the effect and safety of breast reconstruction. However, the case series on complications clearly showed that a BMI equal to or higher than 30 increases the risk for complications by three-fold [32]. Moreover, the most serious complications, such as pulmonary embolism [34] and death [32], occurred in patients with a high BMI. Nonetheless, even though a high BMI clearly increases the risk for complications, there is no evidence to suggest where the exact BMI limit should be. None of the included studies specifically included an analysis of other risk factors for complications, such as smoking [53]. However, the high frequency of wound healing complications in all of the included studies could indicate that all risk factors for wound healing problems should be eliminated.

Regarding effects, breast reduction may improve HRQoL and may reduce depressive symptoms, anxiety symptoms, and pain, compared with no surgery. However, such effect can also be seen when a breast reduction is performed for aesthetic purposes [14, 15, 54]. In this context, it is unclear how such patient-reported outcomes improvement should be valued, and how patients suffering due to appearance related factors should be differentiated from patients with a mere preference for plastic surgery [16, 55]. Moreover, little is known about the long-term effect of plastic surgery on HRQoL, depression and anxiety [56]. In addition, some of the effects, such as the effect of breast reduction on depression, should be interpreted with caution, as the observed baseline values generally indicated no or mild depression [39, 41, 57]. A total of five health economic articles [40, 58–61] were identified in the literature search but only one [40] of them fulfilled the eligibility criteria for inclusion. The studies were all based on studies with small sample sizes, assessing QALY benefits by the intra-individual changes in HRQoL (i.e. lacking control group) and making the optimistic assumption that the HRQoL benefits would last the rest of the lifetime.

The effects seen in this review on HRQoL, depressive symptoms, and anxiety after breast reduction illustrate that breast hypertrophy gives rise to more symptoms than back pain and functional problems, which might indicate that a volume/size requirement is too crude a measure to decide which patients will benefit from a breast reduction and should be granted an operation in the public healthcare system. Moreover, there are no reports on which volumes/weights give rise to physical symptoms in relation to body build and other factors, further strengthening that a volume/size definition, on its own, seems inadequate to predict which patients benefit the most from a breast reduction. In brief, to create evidence-based guidelines for which patients should be granted a breast reduction in the public healthcare system, more studies are needed on the definition of breast hypertrophy and the health care need it gives rise to, as well as on the effect of treatment.

## Conclusions

There are few studies and a lack of high-quality studies that evaluate the results of breast reduction and include a definition of breast hypertrophy. A breast reduction seems to have positive psychological and physical effects for women, but it is unclear which women benefit the most and which women should be offered a breast reduction in the public healthcare system. Currently, there is large variation in, and unequal access to publicly funded breast reduction. A number of priorities for further research have been identified:

- A validated system for how breast hypertrophy should be defined, and which preoperative measurements, symptoms, and outcome variables should be reported in studies
- Evaluation of which volumes/weights give rise to physical symptoms in relation to body build and other factors
- Analysis of what healthcare needs breast hypertrophy gives rise to
- A validated classification system for prospective registration of complications after breast reduction
- Health economical evaluation of the cost-utility of breast reduction compared with no surgery
- Further studies on women’s experiences of breast reduction.

## Supplementary Information

**Additional file 1.** Search strategies.

**Additional file 2.** Excluded studies.

**Additional file 3.** Complications.

**Additional file 4.** Experience of breast reduction.

## Data Availability

The datasets supporting the conclusions of this article are included with the article and its electronic supplements.

## Abbreviations

BDI: Beck’s depression inventory
BMI: Body mass index (kg/m^2^)
CI: Confidence interval
DAS-59: Derriford appearance scale 59
EQ-5D: EuroQol-5 dimension
FBAS: Finnish breast-associated symptoms questionnaire
FPQ: Finnish pain questionnaire
FSFI: Female sexual function index
GRADE: The grading of recommendations assessment, development and evaluation
HADS: Hospital anxiety and depression scale
HAQ-20: Stanford Health Assessment Questionnaire
HTA: Health technology assessment
HRQoL: Health-related quality of life
MD: Mean difference
MID: Minimal important difference
NAC: Nipple areolar complex
NSQIP: The American College of Surgeons National Surgical Quality Improvement Program (ACS NSQIP®)
PICO: P = patients, I = intervention, C = comparison, O = outcome
QALY: Quality adjusted life years
RBDI: Raitsalo’s modification of the BDI (Finnish modification)
RCT: Randomised controlled trial
SBU: Swedish Agency of Health Technology Assessment and Assessment of Social Services
SF-36: Short Form (36) Health Survey
SF-6D: Short Form Six-Dimension
SQoL-F: Sexual quality of life-female
VAS: Visual analogue scale
WMD: Weighted mean difference
