# Supplementary material for: A systematic review and meta-analysis of risks and benefits with breast reduction in the public healthcare system: priorities for further research
Source: BMC Surg. 2021 Sep 11;21:343. doi: 10.1186/s12893-021-01336-7 (PMC8436537; doi:10.1186/s12893-021-01336-7)
Supplement: Supplementary file 1 — Additional file 1. Search strategies. [file 12893_2021_1336_MOESM1_ESM.docx]

**A systematic review and meta-analysis of risks and benefits with breast reduction in the public healthcare system - priorities for further research**

**Emmelie Widmark Jensen, Susanne Bernhardsson, Maud Eriksson, Håkan Hallberg, Christian Jepsen, Lennart Jivegård, Ann Liljegren, Max Petzold, Mikael Svensson, Fredrik Wärnberg, Emma Hansson**

**Electronic supplement 1: Search strategies**

**Database:** Medline (OVID) Ovid MEDLINE(R) ALL 1946 to June 09, 2020

**Date:** 10 June 2020
**No. of results:** 934

| **#** | **Searches** | **Results** |
| --- | --- | --- |
| 1 | ((Hypertrophy or Hypertrophies or Hypertrophied or Hyperplasia* or Asymmetr*) adj6 (Breast or Breasts or Mammary or Mammae or Mammaries)).ab,kf,ti. | 2974 |
| 2 | Hyperplasia/ or Hypertrophy/ | 54032 |
| 3 | exp Breast/ or (Breast or Breasts or Mammary or Mammae or Mammaries).ab,kf,ti. | 487688 |
| 4 | 2 and 3 | 3452 |
| 5 | (Gigantomast* or Macromastia or Hypermastia or Large breast* or Hypertrophic breast*).ab,kf,ti. | 1327 |
| 6 | 1 or 4 or 5 | 6161 |
| 7 | Mammaplasty/ or (Mammaplast* or Mammoplast* or Mastoplast*).ab,kf,ti. | 13845 |
| 8 | (Correct* and (surg* or operative or operation* or procedur*)).ab,kf,ti. | 149990 |
| 9 | (Reduction or Reductions).ab,kf,ti. | 1118093 |
| 10 | 7 or 8 or 9 | 1269430 |
| 11 | 6 and 10 | 1548 |
| 12 | (animals not (animals and humans)).sh. | 4672771 |
| 13 | 11 not 12 | 1513 |
| 14 | (Cancer* or Malign* or Tumor* or Tumour* or Carcinom* or Sarcom* or Neoplasm* or Oncol* or Oncoplast* or oncogen* or Chemotherap* or Chemoradiotherap* or Radiochemotherap* or Chemoradiation or Immunoradiotherap* or Irradiation or Beamtherap* or Radiotherap* or Carcinogen* or Radiation*).ti. | 2283937 |
| 15 | 13 not 14 | 1311 |
| 16 | limit 15 to (yr="1990 -Current" and (danish or english or norwegian or swedish)) | 1004 |
| 17 | (comment or editorial or letter).pt. | 1853775 |
| **18** | **16 not 17** | **934** |

**Database:** PubMed

**Date:** 10 June 2020
**No. of results:** 204

| **Search** | **Query** | **Results** |
| --- | --- | --- |
| **#26** | **Search: #23 AND #24 Filters: Danish, English, Norwegian, Swedish, from 1990 - 2020** | [**204**](https://pubmed.ncbi.nlm.nih.gov/?term=%2323+AND+%2324&filter=language.danish&filter=language.english&filter=language.norwegian&filter=language.swedish&filter=years.1990-2020&sort=relevance) |
| #24 | Search: **(pubmednotmedline[sb] OR inprocess[sb] OR publisher[sb])** Filters: **Danish, English, Norwegian, Swedish** | [4,165,856](https://pubmed.ncbi.nlm.nih.gov/?term=%28pubmednotmedline%5Bsb%5D+OR+inprocess%5Bsb%5D+OR+publisher%5Bsb%5D%29&sort=&filter=language.danish&filter=language.english&filter=language.norwegian&filter=language.swedish) |
| #23 | Search: **#12 AND #18** Filters: **Danish, English, Norwegian, Swedish** | [1,386](https://pubmed.ncbi.nlm.nih.gov/?term=%2312+AND+%2318&filter=language.danish&filter=language.english&filter=language.norwegian&filter=language.swedish&sort=relevance) |
| #19 | Search: **#12 AND #18** | [1,541](https://pubmed.ncbi.nlm.nih.gov/?term=%2312+AND+%2318&sort=) |
| #18 | Search: **#13 OR #16 OR #17** | [1,258,672](https://pubmed.ncbi.nlm.nih.gov/?term=%2313+OR+%2316+OR+%2317&sort=) |
| #17 | Search: **Reduction[Title/Abstract] OR Reductions[Title/Abstract]** | [1,114,634](https://pubmed.ncbi.nlm.nih.gov/?term=Reduction%5BTitle%2FAbstract%5D+OR+Reductions%5BTitle%2FAbstract%5D&sort=) |
| #16 | Search: **#14 AND #15** | [151,523](https://pubmed.ncbi.nlm.nih.gov/?term=%2314+AND+%2315&sort=) |
| #15 | Search: **Surg*[Title/Abstract] OR Operative[Title/Abstract] OR Operation*[Title/Abstract] OR Procedur*[Title/Abstract]** | [3,099,145](https://pubmed.ncbi.nlm.nih.gov/?term=Surg%2A%5BTitle%2FAbstract%5D+OR+Operative%5BTitle%2FAbstract%5D+OR+Operation%2A%5BTitle%2FAbstract%5D+OR+Procedur%2A%5BTitle%2FAbstract%5D&sort=) |
| #14 | Search: **Correct*[Title/Abstract]** | [625,328](https://pubmed.ncbi.nlm.nih.gov/?term=Correct%2A%5BTitle%2FAbstract%5D&sort=) |
| #13 | Search: **Mammaplast*[Title/Abstract] OR Mammoplast*[Title/Abstract] OR Mastoplast*[Title/Abstract]** | [3,857](https://pubmed.ncbi.nlm.nih.gov/?term=Mammaplast%2A%5BTitle%2FAbstract%5D+OR+Mammoplast%2A%5BTitle%2FAbstract%5D+OR+Mastoplast%2A%5BTitle%2FAbstract%5D&sort=) |
| #12 | Search: **#9 or #10** | [9,067](https://pubmed.ncbi.nlm.nih.gov/?term=%239+or+%2310&sort=) |
| #10 | Search: **Gigantomast*[Title/Abstract] OR Macromastia[Title/Abstract] OR Hypermastia[Title/Abstract] OR Large breast*[Title/Abstract] OR Hypertrophic breast*[Title/Abstract]** | [1,321](https://pubmed.ncbi.nlm.nih.gov/?term=Gigantomast%2A%5BTitle%2FAbstract%5D+OR+Macromastia%5BTitle%2FAbstract%5D+OR+Hypermastia%5BTitle%2FAbstract%5D+OR+Large+breast%2A%5BTitle%2FAbstract%5D+OR+Hypertrophic+breast%2A%5BTitle%2FAbstract%5D&sort=) |
| #9 | Search: **#7 and #8** | [7,928](https://pubmed.ncbi.nlm.nih.gov/?term=%237+and+%238&sort=) |
| #8 | Search: **Breast[Title/Abstract] OR Breasts[Title/Abstract] OR Mammary[Title/Abstract] OR Mammae[Title/Abstract] OR Mammaries[Title/Abstract]** | [479,119](https://pubmed.ncbi.nlm.nih.gov/?term=Breast%5BTitle%2FAbstract%5D+OR+Breasts%5BTitle%2FAbstract%5D+OR+Mammary%5BTitle%2FAbstract%5D+OR+Mammae%5BTitle%2FAbstract%5D+OR+Mammaries%5BTitle%2FAbstract%5D&sort=) |
| #7 | Search: **Hypertrophy[Title/Abstract] OR Hypertrophies[Title/Abstract] OR Hypertrophied[Title/Abstract] OR Hyperplasia*[Title/Abstract] OR Asymmetr*[Title/Abstract]** | [323,703](https://pubmed.ncbi.nlm.nih.gov/?term=Hypertrophy%5BTitle%2FAbstract%5D+OR+Hypertrophies%5BTitle%2FAbstract%5D+OR+Hypertrophied%5BTitle%2FAbstract%5D+OR+Hyperplasia%2A%5BTitle%2FAbstract%5D+OR+Asymmetr%2A%5BTitle%2FAbstract%5D&sort=) |

,

**Database:** Embase (OVID) 1974 to 2020 June 09

**Date:** 10 June 2020
**No. of results:** 911

| **#** | **Searches** | **Results** |
| --- | --- | --- |
| 1 | ((Hypertrophy or Hypertrophies or Hypertrophied or Hyperplasia* or Asymmetr*) adj6 (Breast or Breasts or Mammary or Mammae or Mammaries)).ab,kw,ti. | 3720 |
| 2 | exp breast hyperplasia/ | 2532 |
| 3 | exp breast hypertrophy/ | 1237 |
| 4 | (Gigantomast* or Macromastia or Hypermastia or Large breast* or Hypertrophic breast*).ab,kw,ti. | 1659 |
| 5 | 1 or 2 or 3 or 4 | 7301 |
| 6 | exp breast reduction/ | 3015 |
| 7 | (Correct* and (surg* or operative or operation* or procedur*)).ab,kw,ti. | 204212 |
| 8 | (Reduction or Reductions).ab,kw,ti. | 1487672 |
| 9 | (Mammaplast* or Mammoplast* or Mastoplast*).ab,kw,ti. | 4770 |
| 10 | 6 or 7 or 8 or 9 | 1680968 |
| 11 | 5 and 10 | 1859 |
| 12 | (animal not (animal and human)).sh. | 1067242 |
| 13 | 11 not 12 | 1853 |
| 14 | (Cancer* or Malign* or Tumor* or Tumour* or Carcinom* or Sarcom* or Neoplasm* or Oncol* or Oncoplast* or oncogen* or Chemotherap* or Chemoradiotherap* or Radiochemotherap* or Chemoradiation or Immunoradiotherap* or Irradiation or Beamtherap* or Radiotherap* or Carcinogen* or Radiation*).ti. | 2845991 |
| 15 | 13 not 14 | 1529 |
| **16** | **limit 15 to ((danish or english or norwegian or swedish) and yr="1990 -Current" and (article or article in press or conference paper or note or "review"))** | **911** |

**Database:** The Cochrane library

**Date:** 10 June 2020
**No. of results:** 35

*Trials* (35)

| **ID** | **Search** | **Hits** |
| --- | --- | --- |
| #1 | (((Hypertrophy or Hypertrophies or Hypertrophied or Hyperplasia* or Asymmetr*) NEAR/6 (Breast or Breasts or Mammary or Mammae or Mammaries))) | 216 |
| #2 | MeSH descriptor: [Hyperplasia] this term only | 611 |
| #3 | MeSH descriptor: [Hypertrophy] this term only | 602 |
| #4 | #2 OR #3 | 1203 |
| #5 | MeSH descriptor: [Breast] explode all trees | 732 |
| #6 | (Breast or Breasts or Mammary or Mammae or Mammaries):ti,ab,kw (Word variations have been searched) | 47411 |
| #7 | #5 OR #6 | 47418 |
| #8 | #4 AND #7 | 59 |
| #9 | (Gigantomast* or Macromastia or Hypermastia or Large NEXT breast or Hypertrophic NEXT breast):ti,ab,kw (Word variations have been searched) | 80 |
| #10 | #1 OR #8 OR #9 | 310 |
| #11 | MeSH descriptor: [Mammaplasty] this term only | 265 |
| #12 | (Mammaplast* or Mammoplast* or Mastoplast*):ti,ab,kw (Word variations have been searched) | 386 |
| #13 | ((Correct* and (surg* or operative or operation* or procedur*))):ti,ab,kw (Word variations have been searched) | 15378 |
| #14 | Reduction or Reductions | 183549 |
| #15 | #11 OR #12 OR #13 OR #14 | 196997 |
| #16 | #10 AND #15 | 95 |
| #17 | Cancer* or Malign* or Tumor* or Tumour* or Carcinom* or Sarcom* or Neoplasm* or Oncol* or Oncoplast* or oncogen* or Chemotherap* or Chemoradiotherap* or Radiochemotherap* or Chemoradiation or Immunoradiotherap* or Irradiation or Beamtherap* or Radiotherap* or Carcinogen* or Radiation* | 250894 |
| #18 | #16 NOT #17 | 50 |
| #19 | (clinicaltrials or trialsearch):so | 327468 |
| #20 | #18 NOT #19 with Cochrane Library publication date Between Jan 1990 and Jun 2020 | 35 |

**Database:** APA PsycInfo

**Date:** 10 June 2020
**No. of results:** 30

| **#** | **Undran** | **Resultat** |
| --- | --- | --- |
| **S15** | **S12 NOT S13 Avgränsare - Publikationsdatum: 19900101-20200631; Språk: Danish, English, Norwegian, Swedish;** | **30** |
| S14 | S12 NOT S13 | 39 |
| S13 | TI Cancer* or Malign* or Tumor* or Tumour* or Carcinom* or Sarcom* or Neoplasm* or Oncol* or Oncoplast* or oncogen* or Chemotherap* or Chemoradiotherap* or Radiochemotherap* or Chemoradiation or Immunoradiotherap* or Irradiation or Beamtherap* or Radiotherap* or Carcinogen* or Radiation* | 46,067 |
| S12 | S5 AND S11 | 71 |
| S11 | S6 OR S7 OR S10 | 145,466 |
| S10 | S8 AND S9 | 13,232 |
| S9 | TI correct* OR AB correct* OR KW correct* | 136,403 |
| S8 | TI ( surg* or operative or operation* or procedur* ) OR AB ( surg* or operative or operation* or procedur* ) OR KW (surg* or operative or operation* or procedur* ) | 298,295 |
| S7 | TI ( Reduction OR Reductions ) OR AB ( Reduction OR Reductions ) OR KW ( Reduction OR Reductions ) | 132,666 |
| S6 | TI ( Mammaplast* or Mammoplast* or Mastoplast* ) OR AB ( Mammaplast* or Mammoplast* or Mastoplast* ) OR KW ( Mammaplast* or Mammoplast* or Mastoplast* ) | 36 |
| S5 | S3 OR S4 | 1,039 |
| S4 | S1 AND S2 | 67 |
| S3 | TI ( Gigantomast* OR Macromastia OR Hypermastia OR Large breast* OR Hypertrophic breast* ) OR AB ( Gigantomast* OR Macromastia OR Hypermastia OR Large breast* OR Hypertrophic breast* ) OR KW ( Gigantomast* OR Macromastia OR Hypermastia OR Large breast* OR Hypertrophic breast* ) | 976 |
| S2 | TI (Breast or Breasts or Mammary or Mammae or Mammaries) OR AB (Breast or Breasts or Mammary or Mammae or Mammaries) OR KW (Breast or Breasts or Mammary or Mammae or Mammaries) | 18,868 |
| S1 | TI (Hypertrophy or Hypertrophies or Hypertrophied or Hyperplasia* or Asymmetr*) OR AB (Hypertrophy or Hypertrophies or Hypertrophied or Hyperplasia* or Asymmetr*) OR KW (Hypertrophy or Hypertrophies or Hypertrophied or Hyperplasia* or Asymmetr*) | 29,100 |

The web-sites of **SBU** and **Folkehelseinstituttet** were visited

10 June 2020

Nothing relevant to the question at issue was found
