# Supplementary material for: A systematic review and meta-analysis of risks and benefits with breast reduction in the public healthcare system: priorities for further research
Source: BMC Surg. 2021 Sep 11;21:343. doi: 10.1186/s12893-021-01336-7 (PMC8436537; doi:10.1186/s12893-021-01336-7)
Supplement: Supplementary file 2 — Additional file 2. Excluded studies. [file 12893_2021_1336_MOESM2_ESM.docx]

| Aravind, 2020 | Wrong population; unclear BMI |
| --- | --- |
| Blaine, 2012 | Wrong study design, unclear population |
| Chadbourne, 2001 | Unclear population |
| Collins, 2002 | Wrong population; unclear BMI |
| Crittenden, 2020 | Unclear population; BMI <35 not reported separately |
| Crittenden, 2019 | Case series n < 1000 |
| Cruz, 2007 | Wrong outcome |
| Cruz-Korchin, 2004 | Wrong outcome |
| Eggert, 2009 | Case series n < 1000 |
| Fischer, 2014a | Duplicate data set |
| Fischer, 2014b | Duplicate data set |
| Fonseca, 2018 | Wrong comparator, wrong outcome |
| Gust, 2013 | Duplicate data set |
| Iwuagwu, 2005 | Wrong outcome |
| Iwuagwu, 2006a | Wrong outcome |
| Karamanos, 2015 | Wrong population; BMI partially >35 (BMI <35 not reported separately) |
| Kerrigan, 2002 | Wrong population; BMI <35 not reported separately |
| Kordahi, 2015 | Unclear population |
| Lonie, 2019 | Unclear population |
| Manahan, 2015 | Wrong population; BMI <35 not reported separately |
| Mian, 2020 | Unclear population; BMI not reported |
| Nelson, 2014b | Duplicate data set |
| Nuzzi, 2017 | Case series n < 1000 |
| Ovadia, 2018 | Unclear population, wrong comparison |
| Papanastasiou, 2019 | Unclear population |
| Reardon, 2011 | Unclear population; BMI not reported |
| Romeo, 2010 | Wrong comparator; healthy women |
| Saariniemi, 2012 | Wrong study design; cost utility |
| Sharma, 2014 | Case series n <1000 |
| Shermak, 2011 | Unclear population; BMI not reported |
| Singh, 2012 | Unclear population and wrong outcome |
| Taylor, 2004 | Wrong study design; cost effectiveness |
| Thoma, 2014 | Wrong study design; cost effectiveness |
| Tykkä, 2010 | Case series not reporting complications |
| Vairinho, 2018 | Unclear population, wrong intervention and wrong outcome |
| Waltho, 2020 | Wrong outcome, wrong population |
| Vidaeff, 2003 | Wrong study design; case report |
| Woodman, 2007 | Unclear population |
| Zhang, 2016 | Wrong population; BMI <35 not reported separately (also: all case series with <1000 patients, except Nelson 2014 which is already included) |

Aravind P, Siotos C, Bernatowicz E, Cooney CM, Rosson GD. Breast Reduction in Adults: Identifying Risk Factors for Overall 30-Day Postoperative Complications. Aesthet Surg J. 2020;07:07. doi: <https://dx.doi.org/10.1093/asj/sjaa146>.

Blaine CM, Subbio CR, Eid SM, Murphy RX, Jr. Reduction mammaplasty trends: a quality and fiscal analysis update. Ann Plast Surg. 2012;69(4):344-6. doi: 10.1097/SAP.0b013e31824b2707.

Chadbourne EB, Zhang S, Gordon MJ, Ro EY, Ross SD, Schnur PL, et al. Clinical outcomes in reduction mammaplasty: a systematic review and meta-analysis of published studies. Mayo Clin Proc. 2001;76(5):503-10. doi: [10.4065/76.5.503](https://doi.org/10.4065/76.5.503)

Collins ED, Kerrigan CL, Kim M, Lowery JC, Striplin DT, Cunningham B, et al. The effectiveness of surgical and nonsurgical interventions in relieving the symptoms of macromastia. Plast Reconstr Surg. 2002;109(5):1556-66. doi: 10.1097/00006534-200204150-00011.

Crittenden T, Watson DI, Ratcliffe J, Griffin PA, Dean NR, Group AR. Does breast reduction surgery improve health-related quality of life? A prospective cohort study in Australian women. BMJ Open. 2020;10(2):e031804. doi: <https://dx.doi.org/10.1136/bmjopen-2019-031804>.

Crittenden TA, Watson DI, Ratcliffe J, Griffin PA, Dean NR. Outcomes of Breast Reduction Surgery Using the BREAST-Q: A Prospective Study and Comparison with Normative Data. Plast Reconstr Surg. 2019;144(5):1034-44. doi: <https://dx.doi.org/10.1097/PRS.0000000000006114>.

Cruz NI, Korchin L. Lactational performance after breast reduction with different pedicles. Plast Reconstr Surg. 2007;120(1):35-40. doi: 10.1097/01.prs.0000263371.37596.49.

Cruz-Korchin N, Korchin L. Breast-feeding after vertical mammaplasty with medial pedicle. Plast Reconstr Surg. 2004;114(4):890-4. doi: 10.1097/01.prs.0000133174.64330.cc.

Eggert E, Schuss R, Edsander-Nord A. Clinical outcome, quality of life, patients' satisfaction, and aesthetic results, after reduction mammaplasty. Scand J Plast Reconstr Surg Hand Surg. 2009;43(4):201-6. doi: <https://dx.doi.org/10.1080/02844310902891513>.

Fischer JP, Cleveland EC, Shang EK, Nelson JA, Serletti JM. Complications following reduction mammaplasty: a review of 3538 cases from the 2005-2010 NSQIP data sets. Aesthet Surg J. 2014a);34(1):66-73. doi: <https://dx.doi.org/10.1177/1090820X13515676>.

Fischer JP, Wes AM, Kovach SJ. The impact of surgical resident participation in breast reduction surgery--outcome analysis from the 2005-2011 ACS-NSQIP datasets. J Plast Surg Hand Surg. 2014b);48(5):315-21. doi: <https://dx.doi.org/10.3109/2000656X.2014.882345>.

Fonseca CC, Veiga DF, Garcia EDS, Cabral IV, de Carvalho MM, de Brito MJA, et al. Breast Hypertrophy, Reduction Mammaplasty, and Body Image. Aesthet Surg J. 2018;38(9):972-9. doi: <https://dx.doi.org/10.1093/asj/sjx271>.

Gust MJ, Smetona JT, Persing JS, Hanwright PJ, Fine NA, Kim JY. The impact of body mass index on reduction mammaplasty: a multicenter analysis of 2492 patients. Aesthet Surg J. 2013;33(8):1140-7. doi: 10.1177/1090820x13508131.

Iwuagwu OC, Bajalan AA, Platt AJ, Stanley PR, Drew PJ. Effects of reduction mammoplasty on upper-limb nerve conduction across the thoracic outlet in women with macromastia: a prospective randomized study. Ann Plast Surg. 2005;55(5):445-8. doi: 10.1097/01.sap.0000183790.50079.e2.

Iwuagwu OC, Platt AJ, Stanley PW, Hart NB, Drew PJ. Does reduction mammaplasty improve lung function test in women with macromastia? Results of a randomized controlled trial. Plast Reconstr Surg. 2006a;118(1):1-6; discussion 7. doi: 10.1097/01.prs.0000220457.98094.b9.

Karamanos E, Wei B, Siddiqui A, Rubinfeld I. Tobacco Use and Body Mass Index as Predictors of Outcomes in Patients Undergoing Breast Reduction Mammoplasty. Ann Plast Surg. 2015;75(4):383-7. doi: 10.1097/sap.0000000000000192.

Kerrigan CL, Collins ED, Kim HM, Schnur PL, Wilkins E, Cunningham B, et al. Reduction mammaplasty: Defining medical necessity. Med Decis Making. 2002;22(3):208-17. doi: <http://dx.doi.org/10.1177/0272989X0202200309>.

Kordahi AM, Hoppe IC, Lee ES. Reduction Mammoplasty: A Comparison Between Operations Performed by Plastic Surgery and General Surgery. Eplasty [Electronic Resource]. 2015;15:e41.

Lonie S, Sachs R, Shen A, Hunter-Smith DJ, Rozen WM, Seifman M. A systematic review of patient reported outcome measures for women with macromastia who have undergone breast reduction surgery. Gland Surgery. 2019;8(4):431-40. doi: <https://dx.doi.org/10.21037/gs.2019.03.08>.

Manahan MA, Buretta KJ, Chang D, Mithani SK, Mallalieu J, Shermak MA. An outcomes analysis of 2142 breast reduction procedures. Ann Plast Surg. 2015;74(3):289-92. doi: <https://dx.doi.org/10.1097/SAP.0b013e31829d2261>.

Mian S, Dyson E, Ulbricht C. Reduction mammoplasty and back pain: a systematic review and meta-analysis. Eur Spine J. 2020;29(3):497-502. doi: <https://dx.doi.org/10.1007/s00586-019-06155-2>.

Nelson JA, Fischer JP, Wink JD, Kovach SJ, 3rd. A population-level analysis of bilateral breast reduction: does age affect early complications? Aesthet Surg J. 2014;34(3):409-16. doi: <https://dx.doi.org/10.1177/1090820X14525393>.

Nuzzi LC, Firriolo JM, Pike CM, Cerrato FE, Webb ML, Faulkner HR, et al. The Effect of Reduction Mammaplasty on Quality of Life in Adolescents With Macromastia. Pediatrics. 2017;140(5). doi: <https://dx.doi.org/10.1542/peds.2017-1103>.

Ovadia SA, Bishop E, Zoghbi Y, Gasgarth R, Kassira W, Thaller SR. Pedicle De-epithelialization in Reduction Mammoplasty: A Systematic Review of the Literature. Aesthetic Plast Surg. 2018;42(1):100-11. doi: <https://dx.doi.org/10.1007/s00266-017-1024-7>.

Papanastasiou C, Ouellet JA, Lessard L. The Effects of Breast Reduction on Back Pain and Spine Measurements: A Systematic Review. Plastic and Reconstructive Surgery - Global Open. 2019;7(8):e2324. doi: <https://dx.doi.org/10.1097/GOX.0000000000002324>.

Reardon R, Grogan S. Women's reasons for seeking breast reduction: a qualitative investigation. J Health Psychol. 2011;16(1):31-41. doi: <https://dx.doi.org/10.1177/1359105310367531>.

Romeo M, Cuccia G, Zirilli A, Weiler-Mithoff E, Stagno d'Alcontres F. Reduction mammaplasty and related impact on psychosexual function. J Plast Reconstr Aesthet Surg. 2010;63(12):2112-6. doi: 10.1016/j.bjps.2010.01.001.

Saariniemi KM, Kuokkanen HO, Räsänen P, Sintonen H, Tukiainen EJ. The cost utility of reduction mammaplasty at medium-term follow-up: a prospective study. J Plast Reconstr Aesthet Surg. 2012;65(1):17-21. doi: 10.1016/j.bjps.2011.07.028.

Sharma KS, Lim P, Baines R, Brotherston TM. Reduction mammaplasty in adolescents: a review of the indications, timing, and outcomes in a regional plastic surgery unit. Eur J Plast Surg. 2014;37(12):661-6. doi: <http://dx.doi.org/10.1007/s00238-014-1020-y>.

Shermak MA, Chang D, Buretta K, Mithani S, Mallalieu J, Manahan M. Increasing age impairs outcomes in breast reduction surgery. Plast Reconstr Surg. 2011;128(6):1182-7. doi: 10.1097/PRS.0b013e318230c467.

Singh KA, Losken A. Additional benefits of reduction mammaplasty: a systematic review of the literature. Plast Reconstr Surg. 2012;129(3):562-70. doi: <https://dx.doi.org/10.1097/PRS.0b013e31824129ee>.

Taylor AJ, Tate D, Brandberg Y, Blomqvist L. Cost-effectiveness of reduction mammaplasty. Int J Technol Assess Health Care. 2004;20(3):269-73. doi: 10.1017/s0266462304001072.

Thoma A, Kaur MN, Tsoi B, Ziolkowski N, Duku E, Goldsmith CH. Cost-effectiveness analysis parallel to a randomized controlled trial comparing vertical scar reduction and inverted T-shaped reduction mammaplasty. Plast Reconstr Surg. 2014;134(6):1093-107. doi: 10.1097/prs.0000000000000751.

Tykkä E, Räsänen P, Tukiainen E, Asko-Seljavaara S, Heikkilä A, Sintonen H, et al. Cost-utility of breast reduction surgery--a prospective study. J Plast Reconstr Aesthet Surg. 2010;63(1):87-92. doi: 10.1016/j.bjps.2008.08.067.

Vairinho A, Serror K, De Runz A, Noel W, Chaouat M, Mimoun M, et al. Management of nipple-areola complex ischemia after breast reduction: A systematic literature review and algorithm proposal. Eur J Plast Surg. 2018;41(4):369-78. doi: <http://dx.doi.org/10.1007/s00238-018-1399-y>.

Waltho D, Gallo L, Gallo M, Murphy J, Copeland A, Mowakket S, et al. Outcomes and Outcome Measures in Breast Reduction Mammaplasty: A Systematic Review. Aesthet Surg J. 2020;40(4):383-91. doi: <https://dx.doi.org/10.1093/asj/sjz308>.

Vidaeff AC, Ross PJ, Livingston CK, Parks DH. Gigantomastia complicating mirror syndrome in pregnancy. Obstet Gynecol. 2003;101(5 Pt 2):1139-42. doi: 10.1016/s0029-7844(03)00061-9.

Woodman R, Radzyminski S. Women's perception of life following breast reduction: a phenomenological study. Plast Surg Nurs. 2007;27(2):85-92. doi: 10.1097/01.psn.0000278238.10835.55.

Zhang MX, Chen CY, Fang QQ, Xu JH, Wang XF, Shi BH, et al. Risk Factors for Complications after Reduction Mammoplasty: A Meta-Analysis. PLoS ONE [Electronic Resource]. 2016;11(12):e0167746. doi: <https://dx.doi.org/10.1371/journal.pone.0167746>.
