# Supplementary material for: A systematic review and meta-analysis of risks and benefits with breast reduction in the public healthcare system: priorities for further research
Source: BMC Surg. 2021 Sep 11;21:343. doi: 10.1186/s12893-021-01336-7 (PMC8436537; doi:10.1186/s12893-021-01336-7)
Supplement: Supplementary file 3 — Additional file 3. Complications. [file 12893_2021_1336_MOESM3_ESM.doc]

| Freire  2007  Brazil [1] | RCT | 50 | 4 | 8 early complications (n=50, 16%) 1 depressed nipple 6 delayed scar 1 seroma  8 late complications (6 months, n=46, 17%) 6 hypertrophic scars 2 medial dog-ears | Early and late complications were not defined. Patients were evaluated 6 months after surgery. |
| --- | --- | --- | --- | --- | --- |
| Iwuagwu  2006c  UK [2] | RCT | 36 | 0 | 3 major complications (n=36, 8.3%) 2 major wound infections 1 haematoma drained  6 minor complications (6 months, n=16.7%) 6 delayed wound healing | Major and minor complications were not defined. Patients were evaluated 4 months after surgery. |
| Saariniemi  2008  Finland [3] | RCT | 40 | 11 | 4 major complications (n=29, 13.8%) 1 pulmonary embolism 2 haematomas evacuated 1 nipple necrosis  20 minor complications (n=29, 69%) 12 minor infection with opening of wound 4 haematomas  3 ”dog ears”  1 minor nipple necrosis | Complications were not divided into major/minor or early/late. Patients were evaluated 6 months after surgery.  The patient with pulmonary embolism was 57 years old, with a BMI of 32.3, smoked, and had hormone replacement treatment and medication for high blood pressure and hypercholesterolemia, but no thrombosis prophylaxis was given. |
| Janik  2019  Poland [4] | Cohort | 28 |  | 6 complications (21.4%)  5 haematomas  1 nipple necrosis | Complications were not divided into major/minor or early/late. Patients were evaluated 12 to 36 months after surgery. |

**Electronic supplement 3. Complications**

| **Author**  **year**  **country** | **Study design** | **Number of patients n=** | **Withdrawals - drop-outs** | **Results**  **Reduction mammaplasty** | **Comments** |
| --- | --- | --- | --- | --- | --- |

| Fairchild  2020  USA [5] | Case series | 283  BMI<30 | NA | 11 complication in 7 patients (2.5%)  4 surgical site infections  2 30-day-readmissions  3 re-operations  2 wound-dehiscence | NSQIP 2012-2017 register data. NSQIP data are prospectively collected and validated from medical records on preoperative risk factors, preoperative laboratory values, intraoperative variables, 30-day postoperative mortality, and 30-day morbidity. A composite postoperative adverse events variable was created from a list of 21 individual adverse events. Patients were stratified by presence of obesity (body mass index ≥30 kg/m2).  Obesity (BMI≥30) increased the odds of complications by 3-fold (adjusted for operative duration) (p=0.016). In the obese group 1 death and 1 sepsis occurred. |  |  |  |
| --- | --- | --- | --- | --- | --- | --- | --- | --- |
| Nelson  2014  USA [6] | Case series | 2074  BMI<30 | NA | 104 patients had complications (5%)  34 (1.6%) re-operations  48 (2.3%) superficial surgical site infection  6 (0.3%) deep surgical site infections  6 (0.3%) wound dehiscence  5 (0.2%) venous thromboembolism  5 (0.2%) pulmonary embolism  2 (0.1%) unplanned re-intubation  3 (0.1%) urinary tract infection  2 (0.1%) other bleeding | NSQIP 2005-2011 register data. NSQIP data are prospectively collected and validated from medical records on preoperative risk factors, preoperative laboratory values, intraoperative variables, 30-day postoperative mortality, and 30-day morbidity.  Patients were categorised according to the World Health Organisation obesity classification. Data was analysed for surgical complications, wound complications, and medical complications within 30 days of surgery. Surgical complications were defined as an unplanned return to the operating room within 30 days and graft loss or failure. Wound complications included superficial surgical site infections (SSI), deep soft tissue infections, deep organ space infections, and wound dehiscence. Superficial SSI’s were defined as infection involving only skin or subcutaneous tissue of the incision, with purulent drainage and physical exam findings including pain or tenderness, localised swelling, redness, or heat, which was ultimately opened by the surgeon or diagnosed by the surgeon. Deep soft tissue infections were infections involving deeper tissues (muscle or fascia), while organ space infections involve infection of spaces or organs different from that of the incision. Medical complications included any defined NSQIP endpoints such as pneumonia, pulmonary embolism, postoperative renal insufficiency (Creatinine >2 mg/dl), urinary tract infection (UTI), stroke, myocardial infarction (MI), symptomatic deep venous thrombosis (DVT), and sepsis.  BMI > 40 was an independent risk factor for any early complication, OR 2.2 (p<0.001). |  |  |  |
| Simpson  2019  USA [7] | Case series | 8108  BMI<30 | NA | 198 (2.4%) complications | NSQIP 2006-2015 register data. NSQIP data are prospectively collected and validated from medical records on preoperative risk factors, preoperative laboratory values, intraoperative variables, 30-day postoperative mortality, and 30-day morbidity.  Our primary outcome was any major complications within 30 days. Major complication was defined as unplanned readmission or reoperation. Secondary outcomes were defined as wound complications (superficial infection, deep wound infection, deep or organ space infection, and wound dehiscence), medical complications (myocardial infarction, pneumonia, unplanned intubation, urinary tract infection, stroke, pulmonary embolism, deep vein thrombosis, renal insufficiency, sepsis, and death), and requirement for transfusion.  Predictors are not given separately for BMI<30. |  |  |  |

BMI: Body Mass Index (kg/m^2^); NSQIP: The American College of Surgeons' National Surgical Quality Improvement Program; NA: not applicable

1. Freire M, Neto MS, Garcia EB, Quaresma MR, Ferreira LM: **Functional capacity and postural pain outcomes after reduction mammaplasty**. *Plast Reconstr Surg* 2007, **119**(4):1149-1156.

2. Iwuagwu OC, Walker LG, Stanley PW, Hart NB, Platt AJ, Drew PJ: **Randomized clinical trial examining psychosocial and quality of life benefits of bilateral breast reduction surgery**. *Br J Surg* 2006, **93**(3):291-294.

3. Saariniemi KM, Sintonen H, Kuokkanen HO: **The improvement in quality of life after breast reduction is comparable to that after major joint replacement**. *Scand J Plast Reconstr Surg Hand Surg* 2008, **42**(4):194-198.

4. Janik PE, Charytonowicz D, Miszczyk J, Charytonowicz M: **Female Sexual Function and Sexual Well-being Before and After Breast Reduction: A Pilot Cross-sectional Study and Review of Literature**. *Ann Plast Surg* 2019, **82**(6):609-613.

5. Fairchild B, Wei S, Bartz-Kurycki M, Rose JF, Greives MR: **The Influence of Obesity on Outcomes After Pediatric Reduction Mammaplasty: A Retrospective Analysis of the Pediatric National Surgical Quality Improvement Program-Pediatric Database**. *Ann Plast Surg* 2020, **85**(6):608-611.

6. Nelson JA, Fischer JP, Chung CU, West A, Tuggle CT, Serletti JM, Kovach SJ: **Obesity and early complications following reduction mammaplasty: an analysis of 4545 patients from the 2005-2011 NSQIP datasets**. *J Plast Surg Hand Surg* 2014, **48**(5):334-339.

7. Simpson AM, Donato DP, Kwok AC, Agarwal JP: **Predictors of complications following breast reduction surgery: A National Surgical Quality Improvement Program study of 16,812 cases**. *J Plast Reconstr Aesthet Surg* 2019, **72**(1):43-51.
