# Supplementary material for: A systematic review and meta-analysis of risks and benefits with breast reduction in the public healthcare system: priorities for further research
Source: BMC Surg. 2021 Sep 11;21:343. doi: 10.1186/s12893-021-01336-7 (PMC8436537; doi:10.1186/s12893-021-01336-7)
Supplement: Supplementary file 4 [file 12893_2021_1336_MOESM4_ESM.doc]

Author
Year
Country	Study design	Number of patients	Withdrawal - dropouts	Results 	Comments 		Shakespeare and Postle
1999
UK	Qualitative	110






	50/110
(45%)	Advantages:

The majority had very little or no pain (43/60)

Patients reported an increased physical activity. 24 patients felt more fit and 19 patients reported weight loss.

Forty-two patients believed that the operation had changed their lives to the better

Almost all patients were pleased with an increased choice in clothes

Three patients remarked that they believed they had cost the health care system less since the operation

Disadvantages:

Two patients reported a deterioration in self-image and quality of life

One patient expressed regret

Ten patients were unsatisfied with the scaring and expressed discomfort when wearing clothes 
Eight patients were dissatisfied with the nipple (loss of sensibility, misplacement, inversion)

Two patients reported that they experienced more difficulty to interpret mammograms 

Two patients regretted not being able to breastfeed, postpartum, even though they had been informed about this preoperatively.	In-house constructed survey, analysed qualitatively by content analysis

Semi-structured telephone interviews (n=10)
